# Effects of methylphenidate in children with attention deficit hyperactivity disorder: a near-infrared spectroscopy study with CANTAB®

**DOI:** 10.1186/s13034-014-0032-5

**Published:** 2014-12-31

**Authors:** Naomi Matsuura, Makoto Ishitobi, Sumiyoshi Arai, Kaori Kawamura, Mizuki Asano, Keisuke Inohara, Tohru Fujioka, Tadamasa Narimoto, Yuji Wada, Michio Hiratani, Hirotaka Kosaka

**Affiliations:** Tokyo University of Social Welfare, 2-13-32, Marunouchi Naka-ku, Nagoya-City, 460-0002 Japan; Department of Neuropsychiatry, Faculty of Medical Sciences, University of Fukui, 23-3 Matsuoka-Simoaizuki, Eiheiji-cho, Fukui 910-1193 Japan; Research Center for Child Mental Development, University of Fukui, 23-3 Matsuoka-Simoaizuki, Eiheiji-cho, Fukui 910-1193 Japan; Hiratani Pediatric Clinic, Fukui, 1409-2 Kitayotsui, Fukui-shi, Fukui 918-8205 Japan; Department of Child and Adolescent Mental Health, National Institute of Mental Health, National Center of Neurology and Psychiatry, 4-1-1 Ogawa-Higashi, Kodaira, Tokyo 187-8553 Japan; Developmental Emotional Intelligence, Division of Developmental Higher Brain Functions, Department of Child Development United Graduate School of Child Development, 23-3 MatsuokaShimoaizuki, Eiheiji-cho, Fukui 910-1193 Japan; Department of Informatics, Graduate School of Informatics and Engineering, The University of Electro-Communications, 1-5-1 Chofugaoka, Chofu, Tokyo 182-8585 Japan

**Keywords:** Attention Deficit Hyperactivity Disorder (ADHD), Cambridge automated neuropsychological testing battery (CANTAB®), Near-infrared spectroscopy (NIRS), Visuospatial working memory (VSWM), Executive function (EF), Methylphenidate (MPH)

## Abstract

**Background:**

A wide range of evidence supports the methylphenidate (MPH)-induced enhancement of prefrontal cortex (PFC) functioning and improvements in behavioral symptoms in patients with attention deficit hyperactivity disorder (ADHD). Although working memory (WM) has been hypothesized to be impaired in patients with ADHD, no pharmacological studies have examined visuospatial WM (VSWM) with near-infrared spectroscopy (NIRS).

**Study aim:**

The present study was designed to investigate the acute effects of MPH on neuropsychological performance and hemodynamic activation in children with ADHD during VSWM tasks.

**Methods:**

The subject group included 10 boys and 1 girl previously diagnosed with ADHD. Two VSWM tasks of differing degrees of difficulty were conducted. This is the first study on the pharmacological effects of MPH in children with ADHD to evaluate hemodynamic responses in the PFC with simultaneous NIRS.

**Results:**

No significant differences were found in the scores for both spatial working memory (SWM) and score of spatial span (SSP) tasks between the MPH-off and MPH–on conditions. However, a significant MPH-effect on changes in oxy-hemoglobin levels in the PFC was found only in the SWM task.

**Conclusion:**

These findings suggest that PFC activation might be affected by MPH, depending on the degree of difficulty of the particular task. Although the MPH-induced change on behavior may or may not be obvious, NIRS measurements might be useful for assessing the psychological effects of MPH even when performance changes were not observed in the cognitive tasks.

## Introduction

Attention-deficit hyperactivity disorder (ADHD) is a common developmental disorder that affects 3% to 7% of school–age children [1]. The current conceptual models of ADHD are centered on neuropsychological theories of impaired functioning of the frontal lobes, especially the prefrontal cortex (PFC). It has been suggested that the cognitive difficulties that are experienced by children with ADHD are accounted for by deficits in executive functions (EFs) [2]. “EFs” is an overarching term that refers to the mental control processes that enable physical, cognitive, and emotional self-control and that are necessary to maintain effective goal-directed behavior [3]. EFs generally include response inhibition, working memory (WM), cognitive flexibility, planning, and fluency. Among the various EFs, many studies have cited deficits in WM in children and adults with ADHD [4,5]. Kofler et al. suggested that WM is the core and causal cognitive process that is responsible for ADHD in “The Working Memory Model of ADHD” [6].

The term WM refers to a brain system that provides temporary storage and manipulation of the information that is necessary for such complex cognitive tasks as language comprehension, learning, and reasoning [7]. This definition has evolved from the concept of a unitary short-term memory system [7]. WM has been found to require the simultaneous storage and processing of information. It can be divided into the following 3 subcomponents: (i) the central executive, (ii) the visuospatial sketch pad, which manipulates visual images and (iii) the phonological loop [8]. A number of studies have suggested that WM impairments are central to ADHD [8,9]. However, there has been no robust evidence for which component is crucial for the impairment [10]. The most recent findings have indicated that there is growing evidence for impairments in visuospatial WM (VSWM) in patients with ADHD [8,11]. This evidence is consistent with neuropsychological and imaging studies that have mainly implicated right frontal-striatal circuitry impairments in patients with ADHD [12].

Stimulant medications, such as methylphenidate (MPH), are the most commonly prescribed and studied ADHD medications. MPH is highly effective in improving the core symptoms of ADHD [13]. For example, DeVito et al. found that MPH reduced risk-prone betting behavior on the Cambridge Gambling Task in children with ADHD [14]. In a recent review, Pietrzak et al. have found that MPH improved attention control, response inhibition, and sustained attention in approximately 70% of the studies examined [15]. As for WM, it has been reported that MPH also improves WM function by facilitating dopaminergic transmission [16]. Despite these positive effects of MPH on WM [17], there have been a limited number of studies investigating the efficacy of MPH on VSWM in children with ADHD [18]. The objective of the present study was to evaluate the effectiveness of MPH in detail on visuospatial working memory (as well as visuospatial short-term memory) and executive functions of children with ADHD. Therefore, it is very important that the efficacies of medication are assessed in detail from the viewpoint of VSWM because the impairments in VSWM is common to patients with ADHD [11].

The computerized Cambridge Neuropsychological Test Automated Battery (CANTAB®) is one of the most widely used methods to assess EF in pediatric clinical populations [19-22]. The CANTAB® has advantages over other measures of EF because it can be administrated on a computer, (which controls for variations across examiners), has more than 20 subtests for evaluating EF abilities, is nonverbal (requires touch-screen responses), and there is empirical support for the role of prefrontal and medial temporal brain regions in performance on the CANTAB® tasks [23]. It is a suitable battery for children with developmental disorders because of these advantages. The present study employs spatial working (and short-term) memory tasks as well as tasks to assess the executive functions in the CANTAB® in order to evaluate the effectiveness of MPH on those cognitive abilities of children with ADHD. Furthermore, because the CANTAB® includes several VSWM tasks, such as the spatial working memory (SWM) and spatial span (SSP) tasks, it is suitable for evaluating the effectiveness of MPH by implementing these tasks at two condition (MPH-off and MPH–on). For example, Rhodes et al. evaluated the acute neuropsychological effects of MPH in drug-naïve boys with ADHD by conducting many of the CANTAB® subtasks, including the SWM and SSP [24]. They found that MPH did not improve performance on any task. Additionally, there were no significant differences among the baseline, placebo, MPH 0.3 mg/kg, and MPH 0.6 mg/kg groups in scores on visuospatial tasks [24]. Few pharmacological studies have examined VSWM with CANTAB® tasks, and it has been difficult to detect improvements in CANTAB® scores. Additionally, due to the lack of neuroimaging methods in previous research, limited information relating to MPH effects on VSWM has been obtained. Therefore, we set out to examine not only changes in the CANTAB® scores, but also activation of PFC with a brain imaging tool in patients in MPH-on and MPH-off conditions.

Near-infrared spectroscopy (NIRS) is one of the most promising noninvasive functional neuroimaging tools that allows for comparative evaluations of cortical hemodynamic responses in children and individuals with psychiatric disorders. NIRS can measure the signals that reflect relative changes in oxyhemoglobin (oxy-Hb) and deoxyhemoglobin (deoxy-Hb), which are assumed to reflect regional cerebral blood volume. Although functional magnetic resonance imaging and positron emission tomography have many advantages, including high spatial resolution, they have serious limitations in evaluating a drug’s therapeutic effects in bedside settings, especially for children with certain developmental disorders. In contrast, NIRS has many advantages in that it is non-invasive, allows for examination in a natural sitting position, and can be easily attached and removed. In terms of measuring the effects of medications, NIRS is considered the ideal instrument for evaluating prefrontal activation. In fact, several NIRS studies have been performed in children with ADHD during several EF tasks, such as the Stroop Color-Word Task, the Reserve Stroop Task, and the Go/No-go task. Therefore, the pharmacological effects of MPH might be evaluated by changes that are detected in oxy-Hb with NIRS during the course of the performance on specific VSWM tasks of the CANTAB®.

A number of studies have used neurological test batteries, such as the CANTAB®, in children with ADHD, and some of these have suggested that response inhibition performance is improved with MPH compared to placebo. Solanto et al., have evaluated the effectiveness of MPH in 25 children with ADHD using neuropsychological batteries, such as the Continuous Performance Test and the Resistance to Cognitive Interference Test (Stroop Task). They found signifiant effects of MPH on performance on the Continuous Performance Test, but not on performance on the Stroop Task [13]. However, no studies have examined the effects of MPH in children with ADHD by simultaneously measuring the hemodynamic changes of oxy-Hb in prefrontal regions and performance on VSWM tasks. The combination of the CANTAB® tests to evaluate VSWM with NIRS to assess hemodynamic changes in the brain, would provide critical insight into the mechanisms of the effects of MPH. In short, combining the CANTAB® tasks and NIRS evaluations has the potential of elucidating a better understanding of the treatment of children with ADHD.

The present study was designed to investigate the acute effects of MPH on neuropsychological performance and hemodynamic changes in MPH-on and MPH-off conditions in children with ADHD who were performing VSWM tasks of the CANTAB®. Moreover, we examined the relationship between specific EFs, such as VSWM, and behavioral characteristics. To the best of our knowledge, this is the first pharmacological effects study of children with ADHD that has examined performance on CANTAB® tasks and simultaneously evaluated hemodynamic responses in the prefrontal area with NIRS.

## Methods

### Participants

The subject group consisted of 10 boys and 1 girl formally diagnosed with ADHD. The diagnoses were based on the criteria in the Diagnostic and Statistical Manual of Mental Disorders-Version IV-Text Revision [1]. Children were excluded if they had additional disorders such as Pervasive Developmental Disorder, Tourette Syndrome, Obsessive Compulsive Disorder, or Conduct Disorder. Psychiatrists, pediatricians, and other professionals made the final diagnoses. Of twenty initial participants, five children were excluded due to the presence of comorbidities or concurrent medication. Additionally, four children’s NIRS data weren’t analyzed by reason of uncompleted measurement. Finally, eleven participants were involved.

As shown in Table 1, the participants were 10–15 years of age, and their mean age was 10.8 years [standard deviation (SD) = 1.8 years]. The mean scores for full-scale IQ, the verbal comprehension index, the perceptual reasoning index, the working memory index, and the processing index were 102.3 (SD = 17.3), 103.1 (SD = 13.6), 103.4 (SD = 14.6), 98.2 (SD = 23.3), and 98.6 (SD = 12.3), respectively. All participants lived near the Hiratani Pediatric Clinic in Japan and did not receive any public assistance. In addition, no children had experienced parental divorce or child maltreatment, suggesting that they all had similar socio-economic backgrounds.

**Table 1:** **Demographic data for children with treatment**

	**ADHD (N = 11)**
Age M (SD)	10.8 (1.8)
Gender (M/F)	10/1
Full-Scale IQ Mean score (SD)	102.3 (17.3)
Verbal Comprehension Index: VCI Mean score (SD)	103.1 (13.6)
Perceptual Reasoning Index: PRI Mean score (SD)	103.4 (14.6)
Working Memory Index: WMI Mean score (SD)	98.2 (23.3)
Processing Speed Index: PSI Mean score (SD)	98.6 (12.3)
ADHD-RS-IV Total Score Mean score (SD)	69.5 (25.4)
ADHD-RS-IV Inattention Score Mean score (SD)	69.6 (25.4)
ADHD-RS-IV Hyperactivity and Impulsivity score Mean score (SD)	65.4 (26.9)
MPH dose, mg/kg Mean (Range)	33.6 (27–54)
Pubic financial assistance	0
Parental separation	0

### Instruments and neurocognitive tests

#### Japanese version of the home of the ADHD-RS-IV

The ADHD Rating Scale (RS)-IV is an instrument that is reliable, easy-to-administer, and used both for diagnosing ADHD in children and adolescents and for assessing treatment response [25]. The Japanese version of the ADHD-RS was developed by Yamasaki et al. [26] and was has been shown to have good reliability and validity [27]. The participants’ parents completed the ADHD-RS-IV.

#### WISC-IV (Japanese version)

All children completed the 10 Wechsler Intelligence Scale for Children, 4^th^ edition (WISC-IV) which gives 4 summary indices (verbal comprehensive index: VCI, perceptual reasoning index: PRI, working memory index: WMI, and processing speed index: PSI) [28]. The Japanese version of the WISC- IV has been validated in children 5–16 years of age.

#### CANTAB® and conditions

We employed the CANTAB® in order to assess VSWM, which is suggested to be impaired in patients with ADHD. As shown in Table 2, we selected two tasks from the CANTAB®: the SWM in which the core domain is EF, and the SSP in which the core domain is EF.

**Table 2:** **CANTAB tests used in the study and their key output variables**

**Order (Core domain)**	**Sample**	**Domain and associated CANTAB test**	**Test description (Approximate time for Administration)**	**Key measures**
1 (Executive function)		Spatial Working Memory (SWM)	SWM is a test of the participant’s ability to retain spatial information and to manipulate remembered items in working memory. It is a self-ordered task, which also assesses heuristic strategy. This test is a sensitive measure of frontal lobe and ‘executive’ dysfunction. It has been shown in recent studies that impaired performance on SWM emerges as a common factor in prepsychosis (8 min).	#measures for SWM include errors # measure of strategy, and latency measures.
2 (Executive function)		Spatial Span (SSP)	White squares are shown, some of which briefly change colour in avariable sequence. The participant must then touch the boxes which changed colour in the same order that they were displayed by the computer (for clinical mode) or in the reverse order (for reverse mode). The number of boxes increases from 2 at the start of the test to 9 at the end, and the sequence and colour are varied through the test (10 min).	#covering span length (the longest sequence successfully recalled), errors, number of attempts and latency.

Note; The Figures are cited from http://www.cambridgecognition.com/clinicaltrials/cantabsolutions/executive-function-tests.

The SWM is a test of the participant’s ability to retain spatial information and to manipulate remembered items in WM. The test begins with a number of colored squares (boxes) that are shown on the screen. The aim of this test is that, by touching the boxes and using the process of elimination, the participant should find one blue ‘token’ in each of a number of boxes and use them to fill up an empty column on the right hand side of the screen. The number of boxes is gradually increased, until it is necessary to search from a total of 4 to 8 boxes (8 boxes is thought to be very difficult for children).

The SSP, which assesses WM capacity, is a visuospatial analog of the Digit Span test. White squares are shown, some of which briefly change color in a variable sequence. The participant must then touch the boxes that changed color in the same order that they were displayed by the computer (for clinical mode) or in the reverse order (for reverse mode). The number of boxes increases from 2 at the start of the test to 9 at the end, and the sequence and color are varied throughout the test.

**Figure 1:**
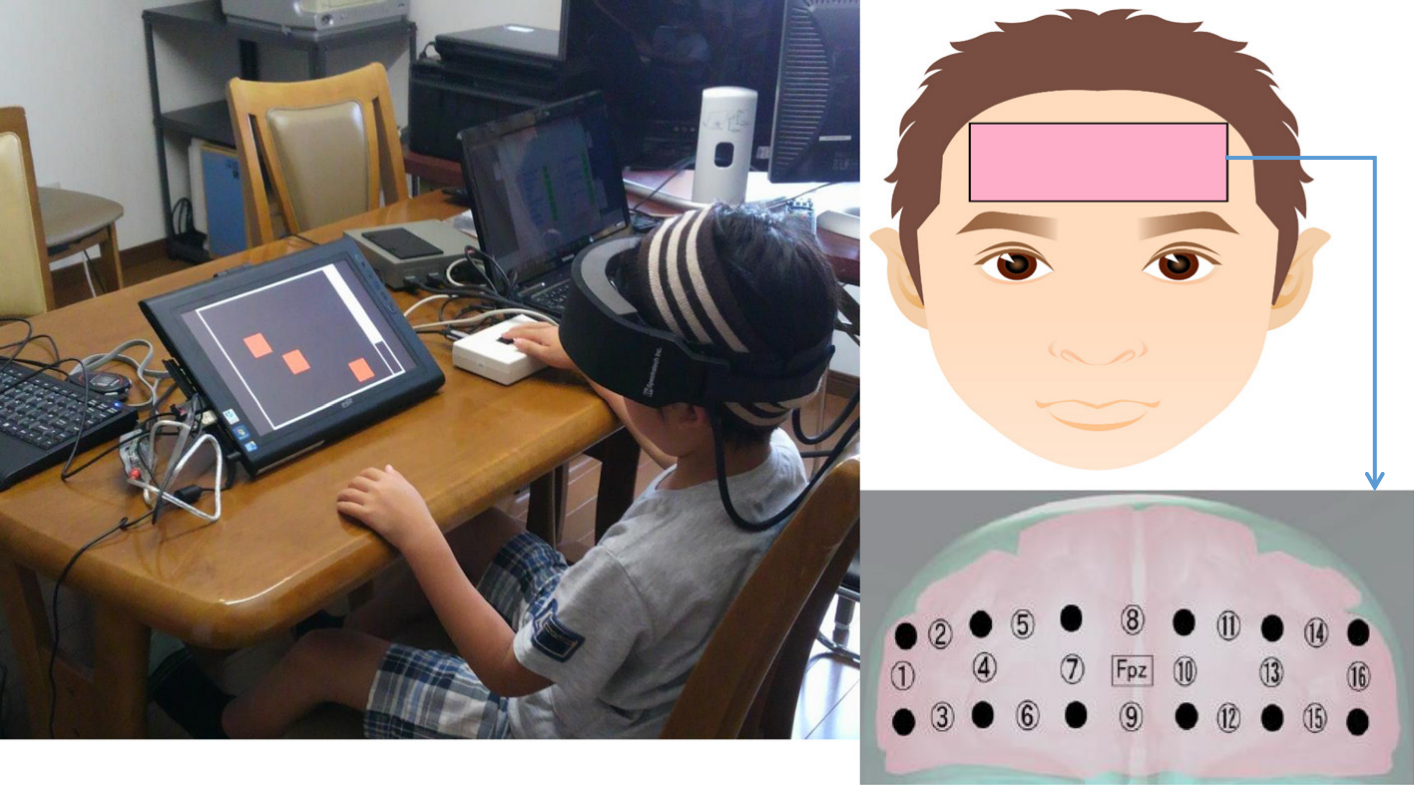
**This picture shows that a subject is conducting CANTAB tasks with an attached the near-infrared spectroscopy (NIRS).** A schematic diagram showing the positoning of NIRS. The NIRS system was attached to the prefrontal area. The center of the probe matrix was placed on Fpz. These figures are cited from DOI:10.1016/j.braindev.2013.01.005. Neurobehavioral and hemodynamic evaluation of Stroop and reverse Stroop interference in children with attention-deficit/hyperactivity disorder.

The order of the two conditions (MPH-off and MPH-on) during CANTAB® tasks was counter balanced across participants. First, before undergoing CANTAB® tests and the WISC-IV, the stimulant medication was withheld for 24 hours, which is a sufficient washout period [29]. About a month later, the second measurements were implemented. If the participants took the drug, the tests were conducted within 4 hours of MPH intake. A primary care doctor in the Hiratani Pediatric Clinic administered the study drug (MPH). As shown in Figure 1, the NIRS instrument was attached to the subject’s head while they performed two tasks.

### NIRS measurement

**Figure 2:**
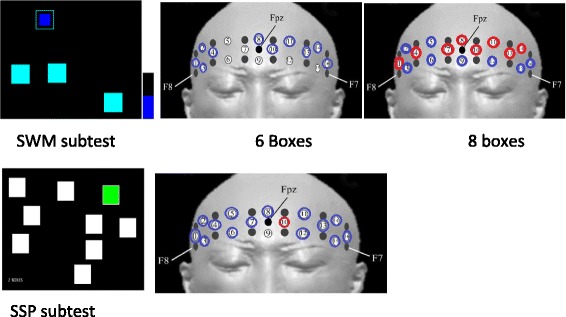
**Hemodynamic changes during performing tasks in MPH-off and –on (blue circle) shows MPH-off > MPH-on, (red circle) shows MPH-off < MPH-on, p < 0.05.** Note; The Figures are cited from http://www.cambridgecognition.com/clinicaltrials/cantabsolutions/executive-function-tests.

Oxy-Hb increases and deoxy-Hb decreases in NIRS have been shown to reflect cortical activation [30]. Because a previous study has revealed that oxy-Hb is more sensitive indicator of brain activation [31], we decided to focus on changes in oxy-Hb. While the participants performed the CANTAB® tasks, neural activity in the PFC was recorded by measuring the changes in oxy-Hb with a multichannel NIRS system (OEG-16; Spectratech Inc., Tokyo, Japan). In this system, near-infrared laser diodes with two different wavelengths (approximately 770 and 840 nm) were used to emit near-infrared light. The re-emitted light was detected with avalanche photodiodes that were located 30 mm from the emitters. The temporal resolution of acquisition was 0.65 s. The system measures oxy-Hb at a depth of approximately 30 mm below the scalp [32]. In this system, 6 emitters and 6 detectors were placed at alternate points on a 2 × 6 grid, enabling us to detect signals from 16 channels (see Figures 1 and 2). The center of the probe matrix was placed on Fpz (International 10–20 system) [33], and the bottom left and bottom right corners were located around F7 and F8, respectively, as in previous studies [30,34].

The measurement principles were based on the modified Beer-Lambert law, for which the [oxy-Hb] was calculated from the changes in light attenuation at a given measurement point. In order to correct for drifting changes in [Hb] over time, each channel was based on two baseline periods: the mean of the 10-second periods before/after the task section. For statistical analyses, we averaged all of the time points of the [oxy-Hb] during the task section. That is, we calculated the [oxy-Hb] using the value of the integral of the data from in each channel in the task.

### Statistical analysis

The mean scores of the CANTAB® subtests were analyzed using paired t-tests (MPH-off and MPH-on). For statistical analysis of the NIRS data, the [oxy-Hb] changes that were detected in each of the 16 channels during the CANTAB® task conditions were included. These NIRS measures, which were the mean [oxy-Hb] from the value of the integral of each channel, were analyzed using paired t-tests (MPH-off and MPH-on). Additionally, we conducted correlation and linear regression analyses in order to examine significant correlations between observed variables. The significance level was set at *p* = 0.05. The analysis was completed with IBM SPSS version 20 (IBM Corporation, Armonk, NY, USA).

### Ethics

The protocol that was used for this study was approved by the ethics committee of the Tokyo University of Social Welfare and the University of Fukui. After a complete explanation of the study, written informed consent was obtained from all subjects and their parents.

## Results

### Demographic data and cognitive, behavioral, and familial characteristics of the participants

The means of the ADHD-RS-IV, the inattention score, and the hyperactive and impulsivity score were 69.5 (SD = 25.4), 69.6 (SD = 25.4), and 69.6 (SD = 25.4), respectively. All children with ADHD received MPH via an osmotic controlled-release oral delivery system (OROS). The mean dose of MPH was 33.6 mg/kg, and the range was 27–54 mg/kg.

### Neuropsychological response to MPH

Table 3 displays the scores from the neuropsychological test batteries for the MPH-on and MPH-off conditions. The results of the SWM (between errors standard score) were −0.05 (SD = 0.76) for MPH-off and 0.00 (SD = 0.85) for MPH-on. There was no significant difference between these two scores [*t* = 0.56, *p* = 0.82]. Similarly, no significant differences were found in the strategy standard score of SWM [*t* = 1.3, *p* = 0.59] and the score in the SSP test [*t* = 1.3, *p* = 0.34].

**Table 3:** **Neuropsychological response to methylhenidate**

	**MPH off**		**MPH on**		**t value** ^**(a)**^	**p**
	**Mean**	**SD**	**Mean**	**SD**		
SWM (Between errors standard score)	−0.05	0.76	0.00	0.85	0.56	0.82
SWM (Strategy standard score)	−0.35	0.77	−0.23	0.72	1.3	0.59
SSP (standard score)	−0.33	0.89	−0.17	0.94	1.3	0.34

Note; ^(a)^pair *t*-test *p < .05.

### Relationships among cognitive ability, SWM and SSP scores, and behavioral performance

**Figure 3:**
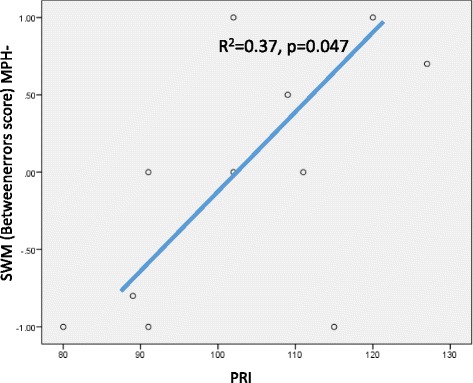
**Relation between PRI and SWM Betweenerrors score.**

Figure 3 indicates that there is a significant correlation between the PRI score, which measures perceptual reasoning abilities, and “the between errors score of SWM” for the MPH-off condition (R^2^ = 0.37, *p* = 0.047). This suggests that a low PRI score is correlated with a higher number of mistakes in the SWM test.

**Figure 4:**
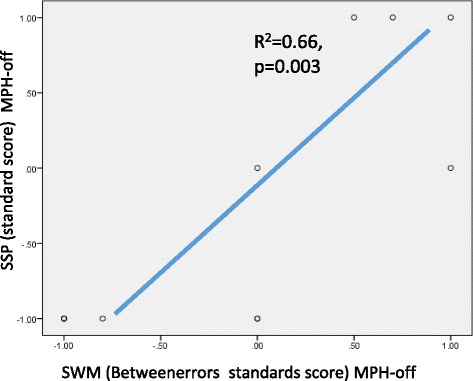
**Rrelation between SWM Betweenerrors standards score and SSP standard score.**

**Figure 5:**
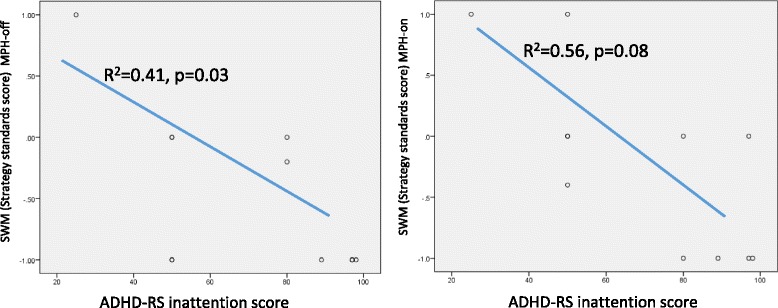
**Relation between SWM strategy standards score and ADHD-RS inattention score.**

Figure 4 shows that “the between errors standard score of SWM” and “the standard score of SSP” were significantly and positively correlated with each other, suggesting a marked linear relationship (R^2^ = 0.66, *p* = 0.003) in the MPH-off condition. However, a significant correlation was not found for these measures in the MPH-on condition. Moreover, the inattention score on the ADHD-RS-IV showed a marked negative correlation with the strategy standard score on the SWM (Figure 5) for both the MPH-off (R^2^ = 0.41, *p* = 0.03) and MPH-on (R^2^ = 0.56, *p* = 0.08) conditions. These data suggest that a tendency towards inattention negatively impacted performance on the SWM and SSP tests.

### NIRS results: hemodynamic changes during task performances for MPH-off and MPH-on conditions

Figure 2 illustrates the patterns of cortical activation measured through oxy-Hb signals in the MPH-off and MPH-on conditions. The inter-condition contrasts (representing the oxy-Hb differences between MPH-off and MPH-on conditions) were statistically examined. In the 6-box subtest of the SWM, a significant MPH effect on oxy-Hb decreases (MPH-off > MPH-on) was found on 10 channels (CHs 1, 2, 3, 4, 8, 10, 12, 13, 14, and 16 paired *t*-test, *p* < 0.05). In addition, in the 8-box subtest, significant increases in oxy-Hb (MPH-off < MPH-on) were seen on 8 channels (CHs 1, 4, 7, 8, 10, 11, 13, and 14 paired *t*-test, *p* < 0.05), and significant decreases in oxy-Hb (MPH-off > MPH-on) were observed on 8 channels (CHs 2, 3, 5, 6, 9, 12, 15, and 16, paired *t*-test, p < 0.05) (Figure 5). In the SSP subtest, a significant MPH effect on oxy-Hb decreases (MPH-off > MPH-on) was detected in all channels except for CH 10 (paired *t*-test, *p* < 0.05) (Figure 2). Although the SWM and SSP scores were not significantly affected by the administration of MPH, effects were clearly observed as changes in brain activation patterns, especially during the SWM tasks.

## Discussion

### Findings from NIRS results employing CANTAB®

To the best of our knowledge, this is the first NIRS study examining the effectiveness of MPH in children with ADHD by measuring hemodynamic responses during a CANTAB® test battery. We focused on tests of EF, especially VSWM, using SWM and SSP tasks. Interestingly, significant differences in hemodynamic responses were observed between the MPH-off and MPH-on conditions. For the 6-box subtest of the SWM, significant oxy-Hb decreases (MPH-off > MPH-on) were found in 10 channels, whereas oxy-Hb was significantly increased in 8 channels (MPH-off < MPH-on) in the 8-box subtest.

Activity in the brain areas that are associated with WM, such as the dorsolateral PFC, has been shown to peak when subjects must maintain upper-limit capacity, decrease under higher-load conditions, and demonstrate an inverted U shape [34,35]. In ADHD subjects, brain activity increases in lower-demand tasks and decreases in tasks in which memory load exceeds capacity [36]. Our findings suggest that the 6-box task might be easier for subjects such that they could operate the task with far less difficulty when administrated MPH. Conversely, the subjects performing the 8-box task, which was considerably more difficult than the 6-box, demanded higher hemodynamics in the PFC due to more intense cognitive processing, which was represented by marked increases of oxy-Hb across wide regions of cortex. In fact, impaired EFs in patients with ADHD become more apparent with increasing task demands [20,37]. However, it cannot be confirmed whether the differential brain activation patterns that were caused by MPH were due to improvements in WM capacity because the brain mechanisms of WM capacity have not been fully studied in subjects with ADHD, and the CANTAB® scores were not changed significantly by the administration of MPH.

Our findings in Figure 4 showed that the SWM and SSP scores were markedly correlated with each other. In addition, no significant changes were found in both the SWM and SSP scores. Therefore, it is noteworthy that there were no significant oxy-Hb increases during the SSP task despite its high level of difficulty. *Why were there no PFC increases in oxy-Hb in the SSP task for the MPH-on condition?* We can examine our findings in detail from the framework of cognitive psychology as follows. The SSP and SWM tasks require temporary retention of spatial information. Unlike the SSP tasks, the SWM tasks require not only temporary retention but also higher cognitive processing. While the SSP tasks require children to temporarily retain visuospatial information, the SWM tasks also require them to effectively look for a target that is hidden in the box. As previous research has shown, children with ADHD have no difficulty in retaining spatial information [18]. Therefore, it is possible that children with ADHD have deficits in the higher cognitive processing of visuospatial information, which is a strategy that is required to successfully complete the task. The pharmacological effects of MPH might be greater on higher cognitive processing. Therefore, after MPH intake, significant oxy-Hb increases were elicited only in SWM tasks. Our findings in the SWM and SSP tasks suggested that MPH effects on PFC activation depende on the degree of difficulty of the task. Therefore, the NIRS measurements can be interpreted even in the absence of significant MPH-based changes in the CANTAB® subtest scores.

### Efficacy of MPH treatment measured with the CANTAB®

Our results showed no significant differences between MPH-off and MPH-on conditions in the scores on the SWM and SSP tests. Because the participants in this study had a normal range of IQs and no comorbidities, MPH intake might not have affected the CANTAB® scores directly. Recently, Biederman et al. have conducted a randomized double-blind study in order to evaluate the association between EF deficits (EFDs) and responses to MPH treatment in patients with ADHD [38]. Their group found that the EFDs did not impact the clinical response to OROS-MPH. These results suggest that EFDs do not determine the response to MPH, and that measures of EFDs are not associated with responses to OROS-MPH. It is unlikely that the CANTAB® scores would be markedly increased to the extent that they would show a significant difference. Although the CANTAB® scores were not changed significantly by the administration of MPH, effects were clearly observed as changes in the brain activation patterns during the SWM tasks. These results suggest that OROS-MPH modulates frontal-lobe function, resulting in clinical responses (improvements) that were not detected by EF tasks. Therefore, we should evaluate not only scores on neurological test batteries, but also hemodynamic changes of oxy-Hb in the PFC in order to assess the efficacy of MPH in detail.

### Relationships between cognitive and behavioral characteristics

We found that the PRI and “between errors score in the SWM” for the MPH-off condition were significantly and positively correlated with each other. Remarkably, the ability to retain spatial information and to manipulate remembered items in WM has a connection with the PRI and not the WMI. The PRI is determined by visual perception, organization, and reasoning abilities with visually presented nonverbal material in order to solve the kinds of problems that are not taught in school [39].

Additionally, the strategy standards score of the SWM were negatively correlated with behavioral data for the ADHD-RS inattention score in both conditions. Own et al. have suggested that an efficient strategy for completing this task is to follow a predetermined sequence by beginning with a specific box, and, then, once a blue token has been found, to return to that box to start the new search sequence [40]. This means that the subjects would require the ability to sustain attention for visuospatial information while executing the effective strategy. An estimate of the use of this strategy has been obtained by counting the number of times that a subject began a new search at the same box [20]. As mentioned earlier, the SWM task simultaneously required temporary retention and active processing. Therefore, subjects who have a tendency for inattention show poor ability to use an effective strategy. In addition, as shown in Figure 2, changes in PFC activity patterns were only observed in the SWM task, which requires more attention compared to the SSP. This might suggest direct or indirect actions of MPH on VSWM.

### Advantages of NIRS measurements while children are conducting the CANTAB® tasks

Although the CANTAB® test battery has been employed in many neurocognitive research studies, as well as in medication efficacy studies, it cannot be implemented in functional magnetic resonance imaging settings. Hence, the activities of the frontal-subcortical circuit have never been described in detail. One strength of our current study was to overcome this technical barrier. NIRS is a useful tool for measuring brain activity, and it is easy to wear. It is very beneficial in that the effectiveness of medications can be evaluated not only by scores on the neurological test battery, but also by the hemodynamic changes of oxy-Hb in the PFC. In recent years, the technology for NIRS measurements has rapidly improved and thus will bring significant benefits to future neuroimaging research.

### Limitations

The current study has a number of limitations that need to be considered. First, the number of participants in the study was not large, and further research is needed to increase the sample size and strengthen the conclusions that can be drawn. Although our intended sample size was twenty, analyzable data were eleven. Second, the participants in this study was not drug-naïve. If the participants was drug-naïve, the effects of MPH on VSWM might be different from those in the present study. Third, because NIRS is unable to detect activity in deep sub cortical structures where near-infrared light cannot reach, the use of the oxy-Hb as a measure of brain area activation was limited to superficial areas. Fourth, because we employed the VSWM tasks of the CANTAB® battery to examine the effects of MPH, combination studies with other EF batteries that specifically focus on VSWM tasks are needed.
